# Clinical significance of matrix metalloproteinase-9 expression in papillary thyroid carcinoma: a meta-analysis

**DOI:** 10.1186/s12957-023-03101-x

**Published:** 2023-07-26

**Authors:** Jinxu Wen, Xiaoru Qin, Jiayi Zhang, Xiaoyong Wu, Xuemin Yan, Kewen Lu, Pei Yang, Shuaichong Ji, Xiangdong Zhao, Yuexin Wang

**Affiliations:** 1 Hebei Medical University, Shijiazhuang, 050051 Hebei Province China; 2 Department of Thyroid and Breast Surgery, Hebei General Hospital, Shijiazhuang, 050051 Hebei Province China; 3 North China University of Science and Technology, Tangshan, 063000 Hebei Province China

**Keywords:** Matrix metalloproteinase-9, Papillary thyroid carcinoma, Immunohistochemistry, Biomarker

## Abstract

**Objective:**

The purpose of this study was to investigate the relationship between the expression of matrix metalloproteinase-9 (MMP-9) and pathological indexes in papillary thyroid carcinoma (PTC).

**Evidence obtained:**

The database was searched in PubMed, Embase, CNKI, and Web of Science databases for relevant clinical trials. The odds ratio (OR) and 95% confidence interval (CI) show the effect of MMP-9 expression and age, tumour size, gender, lymph node metastasis (LNM), and TNM (tumour, lymph node, metastasis) stage. Statistical analysis of the data was performed using Stata 17.0.

**Evidence synthesis:**

A total of 1433 patients with PTC were included in this meta-analysis. MMP-9 expression was significantly correlated with LNM (*OR* = 3.92, 95% *CI* = 2.71–5.65, *P* = 0.000), tumour size (*OR* = 1.69, 95% *CI* = 1.13–2.52, *P* = 0.011), and TNM stage (*OR* = 2.95, 95% *CI* = 2.10–4.13, *P* = 0.000), but not with gender (*OR* = 0.90, 95% *CI* = 0.66–1.22, *P* = 0.487) and age (*OR* = 1.36, 95% *CI* = 0.93–1.98, *P* = 0.115).

**Conclusions:**

Our meta-analysis showed that MMP-9 was significantly associated with LNM, tumour size, and TNM stage; therefore, MMP-9 may be a reliable prognostic biomarker for patients with PTC. However, more high-quality studies are needed to support these findings further.

**Supplementary Information:**

The online version contains supplementary material available at 10.1186/s12957-023-03101-x.

## Introduction

Thyroid carcinoma (TC) is the most common endocrine malignancy, while papillary thyroid carcinoma (PTC) is the most common type of well-differentiated thyroid cancer, accounting for 80–85% of thyroid malignancies [1]. In recent years, with the continuous improvement of high-frequency ultrasound diagnostic technology and the wide application of ultrasound-guided puncture technology, the detection rate of PTC has increased significantly year by year [2]. Although the data suggest a 5-year survival more significant than 97% and 10-year survival of 85% in PTC patients, the odds of relapse at 10 years are about 15% and, at 27 years, about 28% after first treatment [3]. Although the majority of papillary thyroid carcinoma has a good prognosis, factors such as advanced age, male gender, extrathyroidal extension, lymph node or distant metastasis, and high tumour stage have been considered to be poor prognostic factors for many years [4]. Matrix metalloproteinases (MMPs) are zinc-containing endopeptidases from the metzincin family of proteases. MMPs have various physiological functions, which can degrade extracellular matrix (ECM) proteins and glycoproteins, regulate cytokines and growth factors, and participate in embryonic development [5]. The mechanism of action of overexpressed matrix metalloproteinases in malignant tumours is complex and varied. For several years, it has been suggested that the ECM-degrading function of MMPs promotes metastasis and invasion of malignant tumour cells, allowing malignant tumour cells to escape into the surrounding tissues and blood supply [6]. Matrix metalloproteinase-9 (MMP-9), a member of the MMP family, is a critical enzyme to degrade the extracellular matrix. Due to its proteolytic activity, through regulating migration, cancer cell epithelial-mesenchymal transformation, and survival, inducing an immune response, angiogenesis, and the formation of tumour microenvironment plays an essential role in tumourigenesis [7]; moreover, extensive in vitro experiments and in vivo analysis based on animal models have confirmed the role of MMP-9 in tumour development, and much subsequent work has also provided strong evidence for an association between MMP-9 expression and tumour aggressiveness. High expression levels of MMP-9 are associated with the prognosis, diagnosis, and clinical pathology of malignant tumours, such as breast cancer [8], ovarian cancer [9], carcinoma of the lungs [10], and colorectal cancer [11].

Many studies have been published to evaluate the effect of MMP-9 overexpression on clinicopathological features in patients with papillary thyroid carcinoma, such as age, gender, LNM, and TNM stage, but the results of these studies have been inconsistent and contradictory. To more accurately estimate the association between MMP-9 overexpression and clinicopathological features in patients with papillary thyroid carcinoma, we performed a meta-analysis of studies published up to November 2022.

## Materials and methods

### Search strategy

The protocol for this meta-analysis was registered in INPLASY (registration number, INPLASY 202360090) and is available at https://inplasy.com/inplasy-2023-6-0090/. We conducted a comprehensive search in the Embase, PubMed, CNKI, and Web of Science databases for eligible prospective or retrospective cohort studies assessing the prognostic role of MMP-9 overexpression in patients with thyroid cancer. The following search terms were used: (“Thyroid Neoplasms” or “Thyroid Carcinomas” or “Thyroid Cancers” or “Thyroid Adenomas” or “Neoplasm, Thyroid’’) and (“matrix metalloproteinase 9” or “92-kDa Type IV Collagenase” or “Metalloproteinase, MMP-9”). There were no language restrictions, and the last search was updated on Nov 12, 2022. All references cited in these studies were also reviewed to identify additional published articles that were not indexed in the standard database.

### Inclusion and exclusion criteria

The inclusion criteria for the relationship between MMP-9 and thyroid cancer in this meta-analysis are as follows: (1) research must be original and published, (2) all observed patients must be pathologically diagnosed with papillary thyroid carcinoma, (3) determination of MMP-9 expression in tumour tissue, and (4) determination of MMP-9 protein expression rather than mRNA. Exclusion criteria are as follows: (1) Studies’ types were basic studies, case reports, literature reviews, expert opinions, and conference abstracts, (2) papers not published in English or Chinese, (3) overlapping patient cohorts, and (4) non-concomitant effect size.

### Data extraction and quality assessment

Data from eligible studies were evaluated and collected by two authors (Jinxu Wen and Xiaoru Qin): first author, country, year of publication, period, sample size, method, cut-off value, indicator (age, gender, tumour size, LNM, TNM), and Newcastle–Ottawa scale (NOS) score (population selection, comparative score, and outcome score) in Table 1. If the research retrieved cannot be individually classified by title and abstract alone, the full text is reviewed. The two investigators consulted with each other and reached consensus by soliciting the views of a third investigator (Jiayi Zhang) at any disagreement. The quality of selected papers was assessed using the Newcastle–Ottawa scale (NOS). A score of 7–9 indicates good quality, a 6–7 indicates moderate rate, and a score of 5 or below indicates poor quality. Two researchers (Jinxu Wen and Xiaoru Qin) read and evaluated the findings against uniform quality criteria.

**Table 1:** The basic characteristics of the enrolled papers in the study

Author	Year	Country	Period	Method	Cut-off value	Sample size			Indicator	NOS score
						High MMP-9	Low MMP-9	Total		
Li Na [12]	2019	China	2016	IHC	Index ≥ 4	23	17	40	1	7
Wang Ni [13]	2014	China	2011–2012	IHC	Score ≥ 3	48	35	83	1, 2, 3, 4	7
Tian Wei [14]	2017	China	2013–2015	IHC	Score ≥ 3	72	16	88	2, 3, 4, 5	7
Yang Lei [15]	2014	China	2010–2011	IHC	Score ≥ 5	39	33	72	1, 2, 4	6
Meng Xianying [16]	2014	China	2007–2012	IHC	≥ 25%	61	5	66	1, 2, 3, 4, 5	8
Shi Jianhua [17]	2010	China	2002–2006	IHC	> 10%	115	42	157	1	6
Roncevic, J. [18]	2019	Serbia	2000–2015	IHC	NR	29	74	103	1, 2, 4, 5	8
Dong Yuhong [19]	2012	China	2009–2010	IHC	Score ≥ 3	115	53	168	1, 2, 4	6
Wang Zhaohui [20]	2012	China	2012–2010	IHC	> 10%	46	10	56	1, 2, 4	6
Chen Suqin [21]	2018	China	2003–2016	IHC	Score ≥ 3	164	36	200	1, 2, 3, 4	6
Huang Pei [22]	2013	China	2002–2010	IHC	NR	36	22	58	1, 4, 5	6
Liu Xincheng [23]	2020	China	2016–2017	IHC	Score ≥ 4	65	39	104	1, 2, 4, 5	8
Zhang Jie [24]	2010	China	2004–2007	IHC	Score ≥ 3	54	20	74	1	7
Liu Zhen [25]	2014	China	2010–2013	IHC	Score ≥ 3	116	48	164	1, 2, 3, 4, 5	7

Indicator: 1, LNM; 2, TNM; 3, tumour size; 4, gender; 5, age; *NR*, not report in literature

### Statistical analysis

Data analysis was performed using STATA version 17.0 (STATA, College Station, TX, USA). For binary variables, 95% confidence intervals (CI) and odds ratios (OR) were used. Depending on the heterogeneity involved, study results were analysed using either a fixed-effect model or a random-effects model. *P* and *I*^2^ statistics were used for heterogeneity. When *P* was > 0.1 and *I*^2^ < 50%, the fixed-effects (Mantel–Haenszel method) model was used for analysis. When *P* was < 0.1 or *I*^2^ > 50%, the random products (DerSimonian-Laird method) model was used. The causes of statistical heterogeneity were explored through sensitivity analysis; that is, one study was excluded once, and heterogeneity was re-analysed after several other studies were included. In addition, Egger’s and Begg’s tests were used for publication bias. A *P*-value less than 0.05 indicates a statistically significant difference.

## Results

### Description of eligible studies

A total of 745 potentially relevant studies were selected by a preliminary search.

**Fig. 1:**
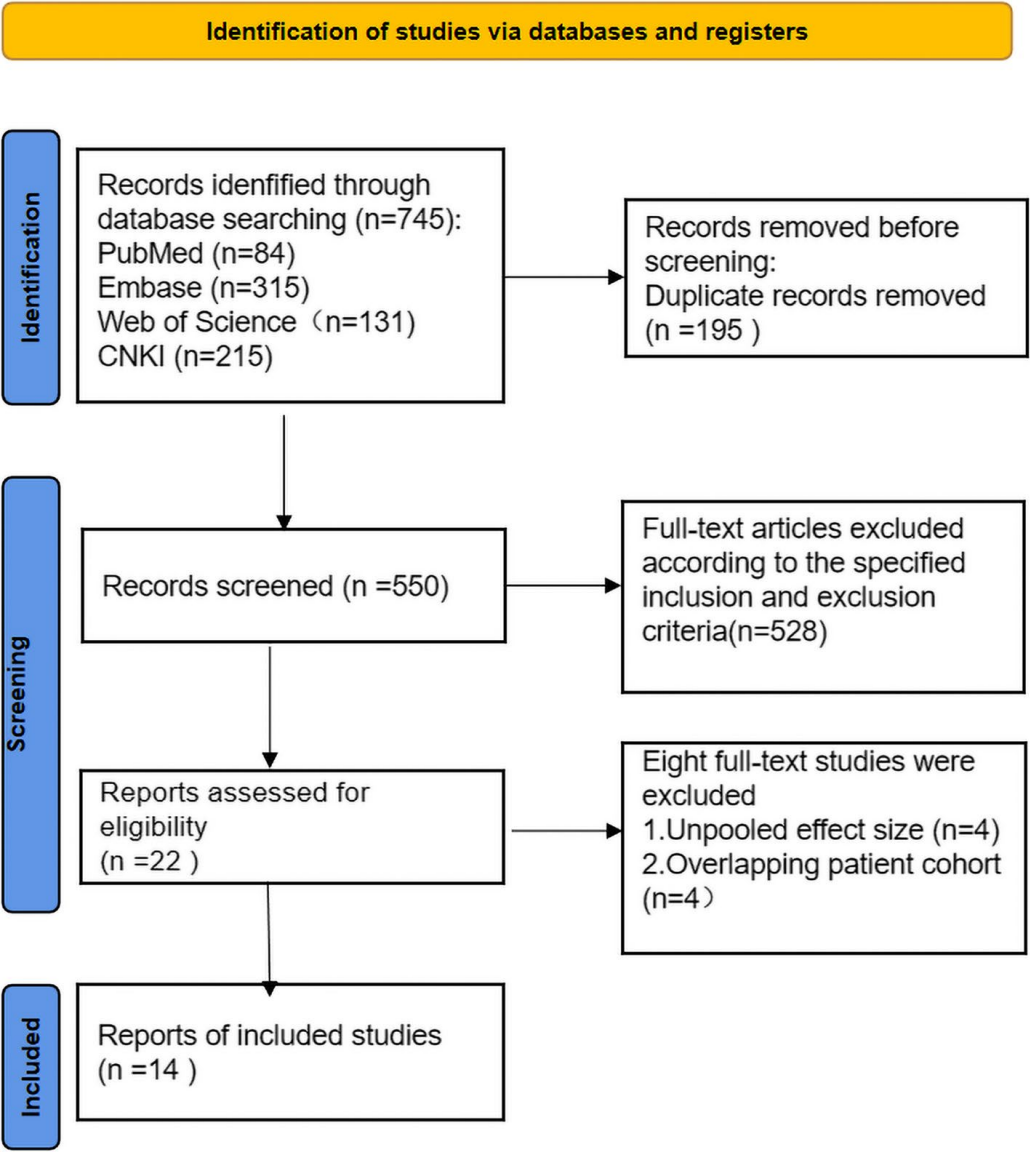
The entire process of literature search has been depicted in the flow diagram

Of these, 723 related studies were excluded because they were comments, basic studies, literature reviews, expert opinions, letters, case reports, or outcomes unrelated relationships with MMP-9 or PTC. After deeply reading the complete text, eight studies were excluded due to patient cohort overlap and non-pooled effect sizes. Finally, we included fourteen published studies [12–25] that reported the correlation between MMP-9 and the clinicopathological indicators of PTC. The entire process involved in searching the literature is described in the flow chart (Fig. 1).

### Study characteristics

**Fig. 2:**
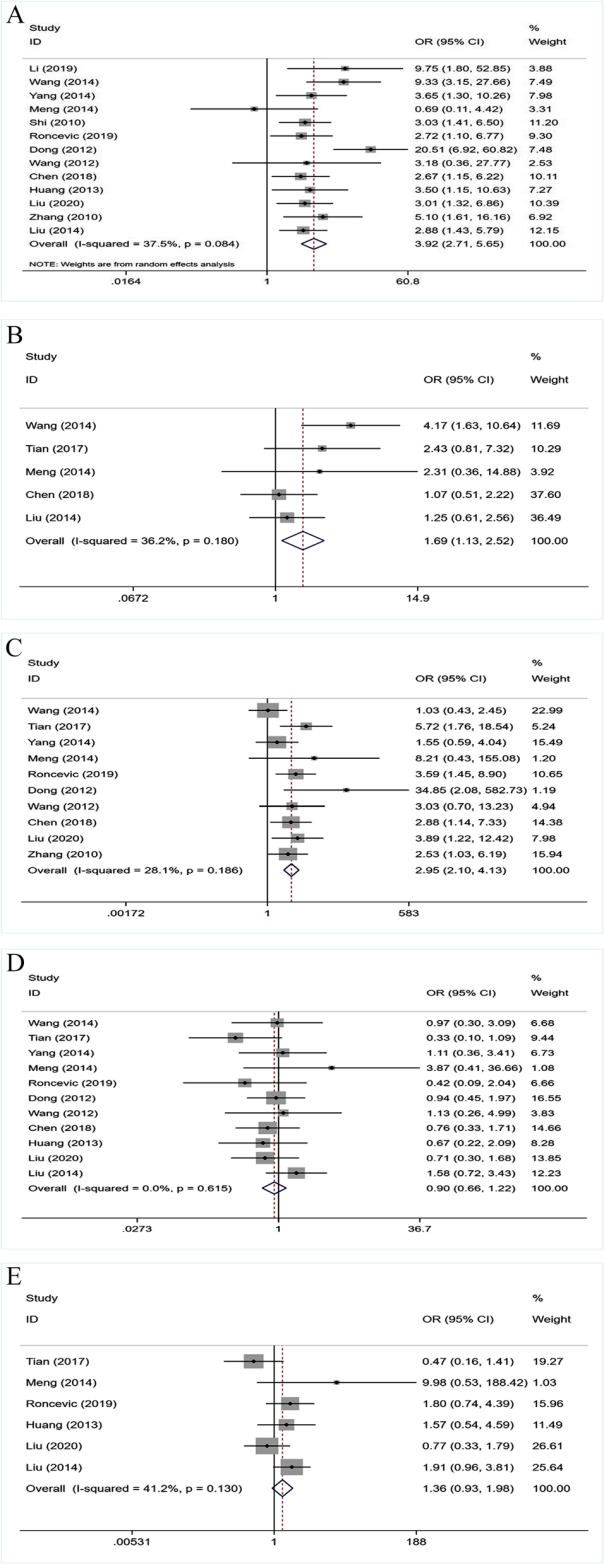
MMP-9 expression in PTC. **A** A total of 1345 PTC patients were collected from thirteen studies to assess whether the expression of MMP-9 in PTC is associated to LNM. **B** A total of 601 female and male patients with PTC were collected from five studies. **C** The OR of the ten studies was pooled, including 1104 stage I/II and stage III/IV. **D** Pooled OR from eleven studies, including 1162 patients with PTC. **E** A total of 578 PTC patients were collected from six studies

A total of 1433 patients with PTC were included in this meta-analysis. The main characteristics of the fourteen eligible studies are summarized in Table 1. Among all the included studies, data of MMP-9 on age, gender, tumour size, LNM, and TNM were reported in six, eleven, five, thirteen, and ten studies, respectively. The correlation between MMP-9 and the clinicopathological features is depicted in Fig. 2.

### Correlation between the expression of MMP-9 and PTC

#### Lymph node metastasis (LNM)

Regarding the cases of lymph node metastases, thirteen studies were included in the meta-analysis. Among 911 patients with MMP-9 expression, 521 (57.2%) had lymph node metastasis, and among 434 patients without MMP-9 expression, 125 (28.8%) had lymph node metastasis. Due to significant heterogeneity in the data, a random-effects model was used (*P* = 0.08, *I*^2^ = 37.5%). According to our analysis, patients with high MMP-9 expression are more likely to develop lymph node metastasis. The overall OR was 3.92 (95% *CI* = 2.71–5.65, *P* = 0.000; Fig. 2A).

#### Tumour size

We included 601 PTC patients from the five studies that reported the tumour size. We used a fixed-effect model because there was no significant heterogeneity between tumour size and MMP-9 expression (*P* = 0.18, *I*^2^ = 36.2%). According to our analysis, patients with high MMP-9 expression had larger tumour volumes than those with low MMP-9 expression (*OR* = 1.69, 95% *CI* = 1.13–2.52, *P* = 0.011; Fig. 2B).

#### TNM stage

Ten studies have investigated the correlation between the expression of MMP-9 and TNM. The overall OR was 2.95 (95% *CI* = 2.10–4.13). No significant heterogeneity in this association was detected using a fixed-effect model (*P* = 0.19, *I*^2^ = 28.1%). Further analysis shows that the advanced TNM stage (III/IV) occurred more frequently in patients with high expression of MMP-9 than in those with low expression of MMP-9 (*P* = 0.000; Fig. 2C).

#### Gender

We screened eleven studies related to gender were included in the meta-analysis. MMP-9 was highly expressed in 206 of 296 male patients (69.6%) and 585 of 866 female patients (67.6%). The overall OR was 0.90 (95% *CI* = 0.66–1.22). No significant heterogeneity of this association was detected using fixed-effects models (*P* = 0.62, *I*^2^ = 0.0%). According to our analysis, MMP-9 expression was not associated with patient gender (*P* = 0.487; Fig. 2D).

#### Age

In total, six age-related studies were selected and included in the meta-analysis. No significant heterogeneity was found by using the fixed-effects model (*P* = 0.13, *I*^2^ = 41.2%). MMP-9 expression was not associated with < 45 years old or ≥ 45 years old (*OR* = 1.36, 95% *CI* = 0.93–1.98, *P* = 0.115; Fig. 2E).

### Sensitivity analysis

**Fig. 3:**
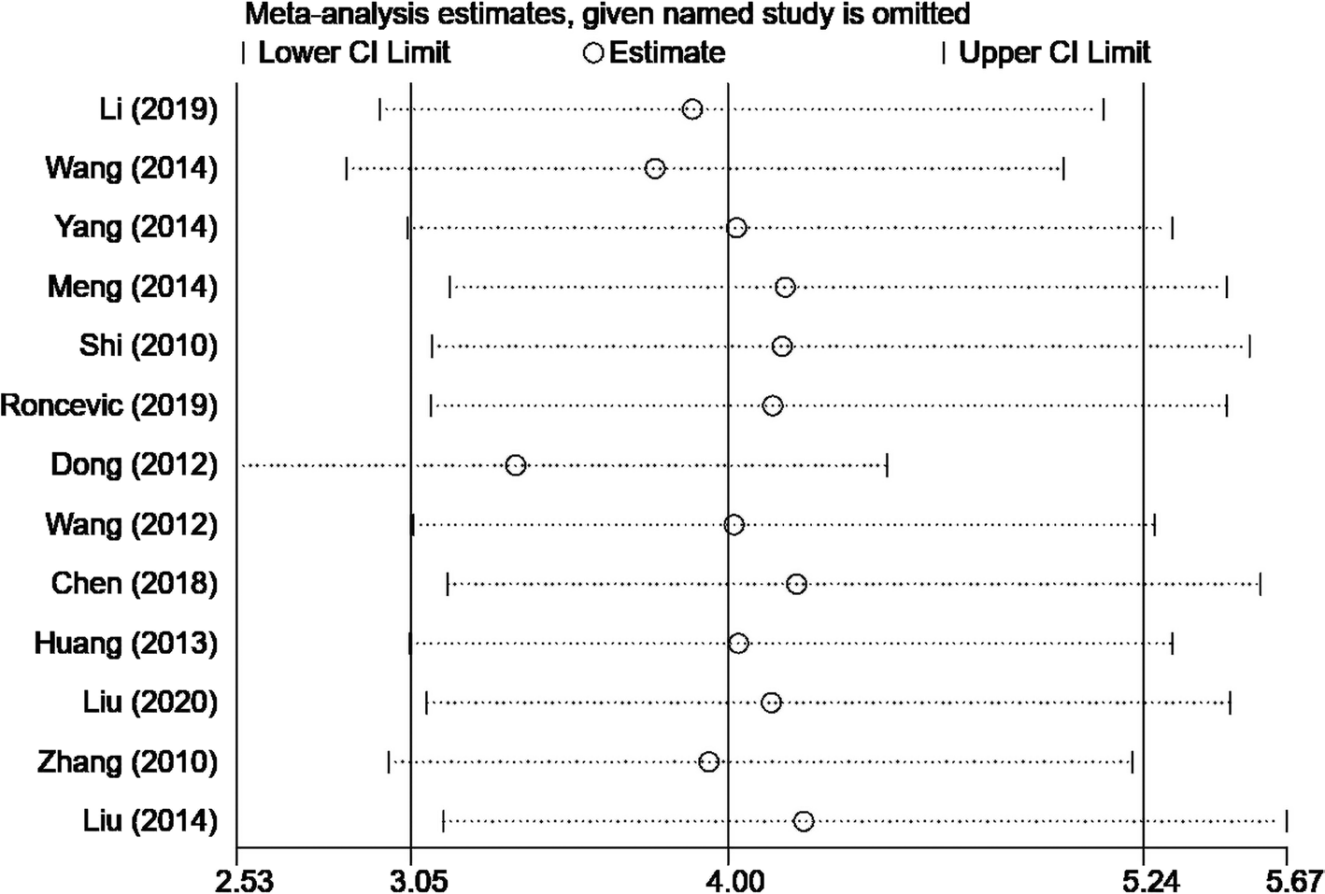
TNM in the sensitivity analysis in the PTC meta-analysis

Heterogeneity exists in the included studies on lymph node metastasis (*P* = 0.06, *I*^2^ = 41.7%). We used the sensitivity analysis to test the stability of this meta-analysis. The analysis was repeated by removing one study at a time, and the exclusion of any study did not significantly change the results, indicating that the results were stable (Fig. 3).

### Publication bias

**Fig. 4:**
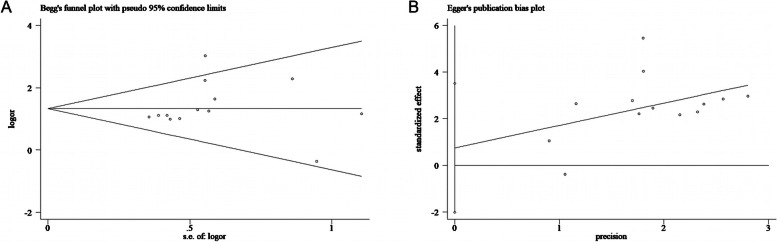
Begg funnel plot and Egger funnel plot in the meta-analysis of PTC. **A** Begg funnel plot of the LNM in the PTC meta-analysis. **B** Egger funnel plot of the LNM in the PTC meta-analysis

To evaluate the stability of the overall estimate, the Begg funnel plot and Egger linear regression test were used to assess bias in this meta-analysis. No publication bias was detected for LNM in Begg’s test (*Pr* >|Z|= 0.246; Fig. 4A) and Egger test (*P* >|*t*|= 0.563; Fig. 4B).

## Discussion

MMP-9, also known as gelatinase B, is one of the critical proteases that degrade the extracellular matrix and basement membrane, which could be activated by other MMPs or tissue plasminogen activator (tPA)-plasmin system, and plays a crucial role in the development of malignancy, invasion, metastasis, and angiogenesis [26, 27]. Previous studies have shown that MMP-9 is overexpressed in multiple cancers and is an important prognostic factor. According to the various studies shown, the positive expression rates of the MMP-9 were significantly higher in PTCs than in normal thyroid tissues [13]; in PTC, tumours were significantly higher than in benign thyroid tumours [16]; and in metastasis, tumour tissues were significantly higher than without metastasis [28]. MMP-9 is not only involved in a series of signaling pathways in thyroid cancer [29–31] but also promote the epithelia-mesenchymal transformation of thyroid carcinoma induced by TGF-1, thereby affecting cell migration and invasion [32]. In contrast to the rapid increase in thyroid cancer incidence, the mortality has remained low and stable over the last decades; balancing treatment risks with risks of disease progression is crucial [33]. Therefore, the identification of more and more molecular biomarkers could not only identify advanced patients who are high risk, more likely to develop metastases and poor prognosis, but also avoid overtreatment of low-risk patients.

A large number of relevant studies have been conducted, but the results are inconsistent due to the existing reports [34–37]. The association of papillary thyroid carcinoma pathological features with MMP-9 remains highly uncertain. This meta-analysis combined the outcomes of 1433 PTC patients from 14 individual studies to investigate the relationship between MMP-9 overexpression and the clinicopathological features of PTC. The results showed that patients with increased MMP-9 expression in PTC tended to have higher TNM stage, larger tumours, and were more prone to lymph node metastasis. In addition, MMP-9 overexpression was not associated with age and gender of PTC patients, which is the same as most studies. Therefore, we have reason to believe that MMP-9 is not only an indicator of patient prognosis but also a possible novel target for the treatment of PTC. No significant publication bias was found in the correlation analysis of this study. Sensitivity analysis showed that risk estimates for all outcomes were not significantly affected by any single study omission. Thus, the results are reliable in the meta-analysis.

In the study of Ilona, M. [38], total MMP-9 immunoreactivity was not associated with any clinicopathological factor, and active MMP-9 was found only in tumour tissue and was significantly associated with age, lymph node metastasis, extrathyroid infiltration, and degree of tumour infiltration; they suggested that overexpression of MMP-9 alone would not lead to the more aggressive behaviour of PTC. However, in the study of Roncevic, J. [18], the overexpression of MMP-9 was correlated with tumour size, TNM stage, lymph node metastasis, extrathyroid invasion, and degree of tumour invasion; active MMP-9 is associated only with lymph node metastasis and extrathyroid infiltrates. Although our meta-analysis confirmed the correlation between the overexpression of MMP-9 and the clinicopathology of PTC, little is known about the relationship between active MMP-9 and PTC, which needs to be confirmed by more relevant studies in the future.

Although we included all potentially eligible data to summarize the clinicopathological features of MMP-9 overexpression in thyroid papillary carcinoma, there were several confounding factors in this study that could affect the stability of our conclusions. Firstly, most of the literature included in this meta-analysis is from China. The results may only apply to Chinese or Asian populations, and publication bias is almost inevitable, and more reports from other regions need to be included to support this meta-analysis. Secondly, the experimental methods of the articles included in this study were all immunohistochemical analysis, and the results may be affected by the antibody used, antibody concentration, storage time, fixation method of paraffin-embedded tissue, and critical value. Finally, with the few articles and patients in this meta-analysis, more high-quality, multiple-sample size studies are still needed to support this result.

## Conclusion

Although this paper has some limitations, the study’s results are still meaningful. From our main analysis results, we found that MMP-9 was not correlated with age and gender but was significantly correlated with tumour size, LNM, and TNM. The high expression of MMP-9 may be a reliable biomarker of poor prognosis in patients with thyroid papillary carcinoma, which can be used for future research and clinical diagnosis. In addition, it could be a potential target for cancer treatment. However, more high-quality, large-sample size studies and randomized controlled trials are needed in the future.

## Supplementary Information

**Additional file 1:** **Supplementary file 1.** The detailed search strategies for each database.

## Data Availability

The data that support the findings of this study are available from the corresponding author upon reasonable request.

## Abbreviations

MMP: Matrix metalloproteinase
PTC: Papillary thyroid carcinoma
TC: Thyroid carcinoma
CNKI: China National Knowledge Infrastructure
LNM: Lymph node metastasis
ECM: Extracellular matrix
TGF-β1: Transforming growth factor-β1
OR: Odds ratio
CI: Confidence interval
