# Supplementary material for: Clinical significance of matrix metalloproteinase-9 expression in papillary thyroid carcinoma: a meta-analysis
Source: World J Surg Oncol. 2023 Jul 26;21:225. doi: 10.1186/s12957-023-03101-x (PMC10369753; doi:10.1186/s12957-023-03101-x)
Supplement: Supplementary file 1 — Additional file 1: Supplementary file 1. The detailed search strategies for each database. [file 12957_2023_3101_MOESM1_ESM.doc]

**Supplementary file 1. The detailed search strategies for each database.**

1. **Search strategies for PubMed:**

(("Matrix Metalloproteinase 9"[Mesh]) OR ((((((((((((Metalloproteinase 9, Matrix[Title/Abstract]) OR (92-kDa Type IV Collagenase[Title/Abstract])) OR (92 kDa Type IV Collagenase[Title/Abstract])) OR (Matrix Metalloproteinase-9[Title/Abstract])) OR (MMP9 Metalloproteinase[Title/Abstract])) OR (Metalloproteinase, MMP9[Title/Abstract])) OR (MMP-9 Metalloproteinase[Title/Abstract])) OR (MMP 9 Metalloproteinase[Title/Abstract])) OR (Metalloproteinase, MMP-9[Title/Abstract])) OR (92-kDa Gelatinase[Title/Abstract])) OR (92 kDa Gelatinase[Title/Abstract])) OR (Gelatinase B[Title/Abstract]))) AND ((("Thyroid Neoplasms"[Mesh]) OR ((((((((((((((((((Neoplasm, Thyroid[Title/Abstract]) OR (Thyroid Neoplasm[Title/Abstract])) OR (Neoplasms, Thyroid[Title/Abstract])) OR (Thyroid Carcinoma[Title/Abstract])) OR (Carcinoma, Thyroid[Title/Abstract])) OR (Carcinomas, Thyroid[Title/Abstract])) OR (Thyroid Carcinomas[Title/Abstract])) OR (Cancer of Thyroid[Title/Abstract])) OR (Thyroid Cancers[Title/Abstract])) OR (Thyroid Cancer[Title/Abstract])) OR (Cancer, Thyroid[Title/Abstract])) OR (Cancers, Thyroid[Title/Abstract])) OR (Cancer of the Thyroid[Title/Abstract])) OR (Thyroid Adenoma[Title/Abstract])) OR (Adenoma, Thyroid[Title/Abstract])) OR (Adenomas, Thyroid[Title/Abstract])) OR (Thyroid Adenomas[Title/Abstract])))))

**2. Search strategies for Web of Science:**

1: ((((((((((((TS=(Matrix Metalloproteinase 9)) OR TS=(Metalloproteinase 9, Matrix)) OR TS=(92-kDa Type IV Collagenase)) OR TS=(92 kDa Type IV Collagenase)) OR TS=(Matrix Metalloproteinase-9)) OR TS=(MMP9 Metalloproteinase)) OR TS=(Metalloproteinase, MMP9)) OR TS=(MMP-9 Metalloproteinase)) OR TS=(MMP 9 Metalloproteinase)) OR TS=(Metalloproteinase, MMP-9)) OR TS=(92-kDa Gelatinase)) OR TS=(92 kDa Gelatinase)) OR TS=(Gelatinase B)

2: (((((((((((((((((TS=(Thyroid Neoplasms)) OR TS=(Neoplasm, Thyroid)) OR TS=(Thyroid Neoplasm)) OR TS=(Neoplasms, Thyroid)) OR TS=(Thyroid Carcinoma)) OR TS=(Carcinoma, Thyroid)) OR TS=(Carcinomas, Thyroid)) OR TS=(Thyroid Carcinomas)) OR TS=(Cancer of Thyroid)) OR TS=(Thyroid Cancers)) OR TS=(Thyroid Cancer)) OR TS=(Cancer, Thyroid)) OR TS=(Cancers, Thyroid)) OR TS=(Cancer of the Thyroid)) OR TS=(Thyroid Adenoma)) OR TS=(Adenoma, Thyroid)) OR TS=(Adenomas, Thyroid)) OR TS=(Thyroid Adenomas)

3: #2 AND #1

1. **Search strategies for Embase:**

#1. 'gelatinase b'/exp

#2. 'metalloproteinase 9, matrix':ab,ti OR '92-kda type iv collagenase':ab,ti OR '92 kda type iv

collagenase':ab,ti OR 'matrix metalloproteinase-9':ab,ti OR 'metalloproteinase, mmp9':ab,ti OR 'mmp9 metalloproteinase':ab,ti OR 'mmp-9 metalloproteinase':ab,ti OR 'mmp 9 metalloproteinase':ab,ti OR 'metalloproteinase, mmp-9':ab,ti OR '92-kda gelatinase':ab,ti OR '92 kda gelatinase':ab,ti OR 'matrix metalloproteinase 9':ab,ti

#3. #1 OR #2

#4. 'thyroid cancer'/exp

#5. 'thyroid neoplasms':ab,ti OR 'neoplasm, thyroid':ab,ti OR 'thyroid neoplasm':ab,ti OR

'neoplasms, thyroid':ab,ti OR 'carcinoma, thyroid':ab,ti OR 'carcinomas, thyroid':ab,ti OR 'thyroid carcinomas':ab,ti OR 'cancer of thyroid':ab,ti OR 'thyroid cancers':ab,ti OR 'cancer, thyroid':ab,ti OR 'cancers, thyroid':ab,ti OR 'cancer of the thyroid':ab,ti OR 'adenoma, thyroid':ab,ti OR 'adenomas, thyroid':ab,ti OR 'thyroid adenomas':ab,ti

#6. #4 OR #5

#7. #3 AND #6
